# Diagnostic value of open incisional biopsies in suspected, difficult-to-diagnose periprosthetic hip joint infection prior to revision surgery

**DOI:** 10.1007/s00402-022-04402-8

**Published:** 2022-03-28

**Authors:** M. J. K. Simon, J. Beyersdorff, A. Strahl, T. Rolvien, W. Rüther, Andreas Niemeier

**Affiliations:** 1 Department of Orthopaedics, University Medical Center Hamburg-Eppendorf, Martinistrasse 52, 20246 Hamburg, Germany; 2 Department of Orthopaedics and Trauma Surgery, University Medical Center Schleswig-Holstein, Arnold-Heller-Str. 3, 24105 Kiel, Germany

**Keywords:** Periprosthetic joint infection, Hip arthroplasty, Revision surgery, Microbiology, Histology

## Abstract

**Introduction:**

Prior to revision of total hip arthroplasty (THA), low-grade chronic periprosthetic joint infection (PJI) is often difficult to diagnose. We aimed to determine the diagnostic accuracy of open incisional tissue biopsy for the prediction of PJI prior to THA revision in cases with culture-negative or dry tap joint aspirates.

**Materials and methods:**

This retrospective single-center study includes 32 consecutive THA revision cases with high clinical suspicion of low-grade chronic PJI of the hip with culture-negative or dry tap joint aspirates and without systemic signs of infection. Open incisional biopsy (OIB) was performed prior to revision surgery. Periprosthetic tissue samples were analyzed by microbiology and histopathology for PJI. During definitive revision arthroplasty, identical diagnostics were repeated. Results from both procedures were compared and sensitivity, specificity, positive and negative predictive values of OIB for the final diagnosis were calculated.

**Results:**

Average age at revision was 69.3 ± 13.5 years. The sensitivity of the OIB procedure was 80% (microbiology), 69% (histology) and 82% for combined analyses (microbiology and histology). Specificity of OIB was 80% (microbiology), 94% (histology) and 60% for combined analyses.

**Conclusions:**

Open tissue biopsy performed in cases with culture-negative or inconclusive synovial fluid aspirates prior to revision of THA has limited diagnostic accuracy for the prediction of PJI. The procedure does not reliably close the diagnostic gap in a substantial number of cases. In this difficult patient population, risk of an open procedure may outweigh benefits and alternative less invasive methods should be considered for the preoperative diagnosis of PJI.

## Introduction

Periprosthetic joint infection (PJI) is a severe complication of total hip arthroplasty (THA) and one of the major reasons for THA revision [14, 34]. Chronic low-grade infections can represent a diagnostic challenge [11, 16, 18, 21, 28]. In cases with negative microbiology from synovial fluid but a clinical suspicion of PJI, periprosthetic tissue biopsies can be taken in an effort to establish the diagnosis of PJI [28]. Knowledge of the pathogens and their resistance prior to revision is of relevance for the operative strategy (one stage versus two stage, addition of specific antibiotics to the bone cement) and is crucial for outcome.

To best of the authors’ knowledge, the diagnostic value of incisional biopsies has not been described in this problematic patient subpopulation.

There are several options of taking tissue biopsies: by open incision, by arthroscopy, by mini incision without arthroscopic assistance with or without fluoroscopic guidance [3, 12, 24, 28].

Although open biopsy has the potential advantage of better visualization and controlled access to different intra-articular localizations, in particular at the prosthesis tissue interface [3], it is unknown whether open incisional biopsy has a diagnostic advantage over the less invasive methods of tissues sampling.

In addition, it is unknown to which degree microbiology findings from biopsies predict microbiology results from definitive revision surgery with either method of tissue sampling in this problematic subgroup of patients.

The aim of the present study was to describe the diagnostic accuracy of open tissue biopsy in patients with scheduled THA revision surgery, a clinical suspicion of low-grade chronic PJI but negative microbiology from synovial joint aspirates.

## Materials and methods

This retrospective analysis was performed at one academic arthroplasty center and included 32 consecutive cases (2013–2017) with suspected chronic low-grade THA PJI despite negative synovial fluid microbiology.

Suspected chronic low-grade THA PJI was defined as unexplained prolonged loco-regional pain (> 3 weeks), with or without radiographic signs of PJI such as subtle osteolysis, otherwise unexplained increased systemic serum CRP > 1 mg/dl and/or a WBC in synovial fluid of > 2000/µl [23].

Prior to hip revision arthroplasty, every patient received a pre-operative fluoroscopy guided synovial fluid aspiration from anterolateral or superolateral. In case of positive cultures, patients underwent septic two-stage revision. In case of negative cultures, and no further suspicion of a PJI (clinical, radiological or blood work), a single-stage presumably aseptic revision surgery was performed with intra-operative single shot antibiotics once all tissue specimens had been taken. In case of negative cultures but a clinical, radiographic or laboratory-based suspicion of chronic PJI, an open incisional biopsy (OIB) was performed.

Inclusion criteria for the present study thus were (i) clinical suspicion of PJI (ii) negative microbiological findings from synovial fluid or a dry tap (no synovial fluid available for microbiology).

Six cases had a dry tap and in 26 cases less than 0.5 ml synovial fluid was aspirated, which was sent for microbiological analysis and returned negative. Analysis of further synovial fluid parameters such as WBC count and others was not performed due to the limited amount of synovial fluid.

**Fig. 1:**
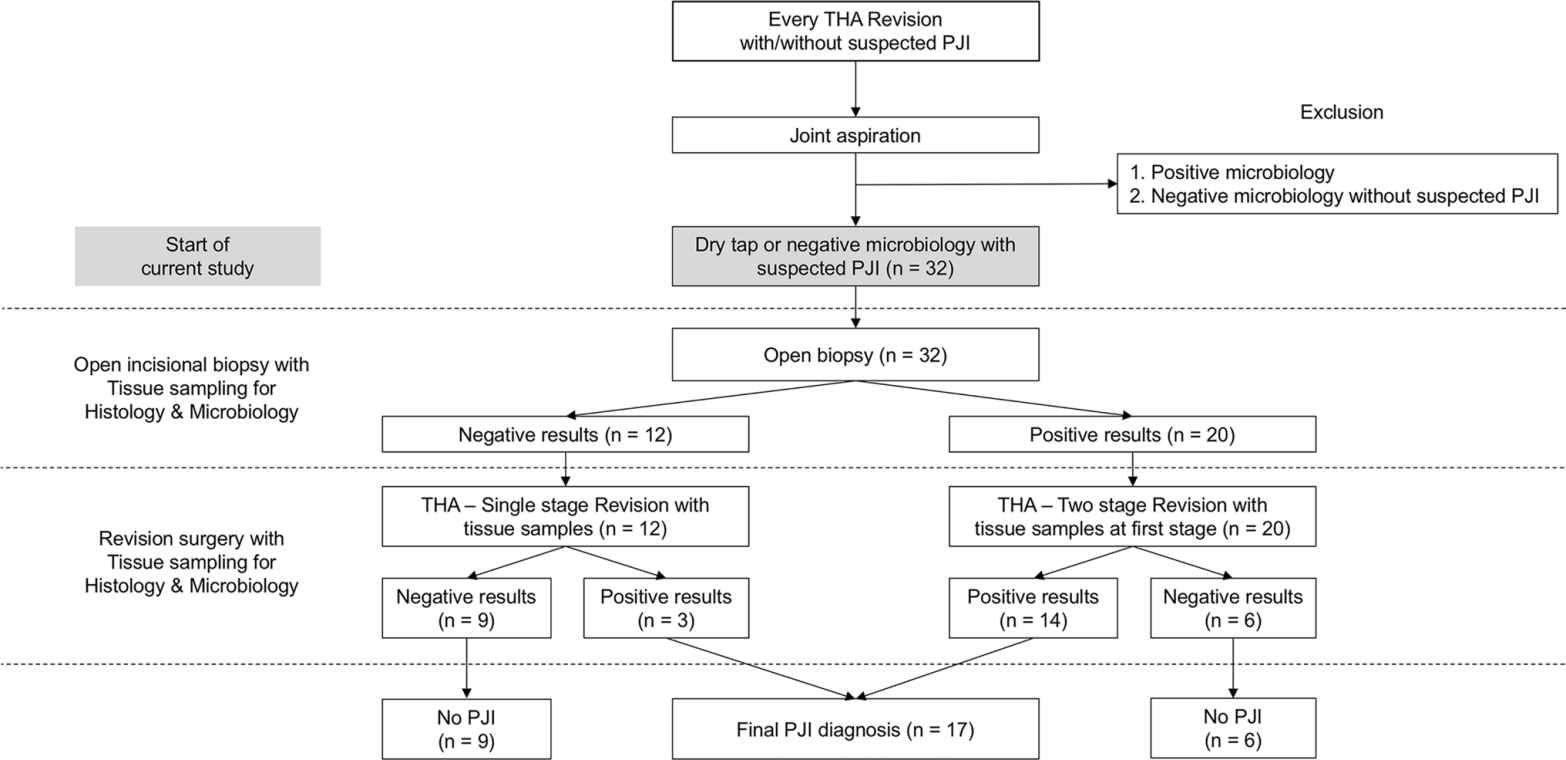
Flowchart demonstrates included total hip arthroplasty (THA) cases and different sampling stages (open incisional biopsy and revision surgery) with the positive or negative results with regard to periprosthetic joint infections (PJI)

There were no signs of local inflammation concerning the skin, e.g., swelling, erythema, warmth, or fistulae in these patients. All cases in which the biopsy led to the diagnosis of PJI were then treated by a septic two-stage revision with an antibiotic treatment regimen according to the susceptibility of the detected pathogens [6, 21] (Fig. 1, flowchart).

OIB is a separate procedure prior revision surgery. The procedure is carried out in a sterile fashion in the operating theater. A smaller than standard modified posterior hip approach (Moore) is used to expose the region of interest (hip prothesis) and obtain periprosthetic tissue samples.

Culture time for all samples was 14 days. Samples without growth after 14 days in culture were regarded negative and discarded. The patient was informed about culture results on average 3 weeks after OIB, and revision surgery was scheduled an average 6–8 weeks after OIB.

During revision surgery, periprosthetic tissue sampling for microbiology and histology analyses were repeated, and the results of these were defined as the definite diagnosis.

Biopsy and revision procedures were all carried out in sterile conditions in an operating theater. A modified posterior hip approach was used for all procedures, i.e., OIBs (mini open) and revisions (extended approach including previous OIB incision). A minimum of five periprosthetic tissue biopsy samples (capsular and its surrounding tissue, acetabular and femoral component surrounding tissue, and other tissues, particularly abnormal appearing tissue) for microbiology and a minimum of one tissue sample for histology were obtained at each surgical procedure, similar to a previous report reporting on tissue sampling during revision surgery [3]. None of the patients were given antibiotics for 4 weeks prior to biopsy or revision surgeries as previously described [10, 11, 22].

Intraoperative ###samples were placed into sterile bottles containing culture-enrichened thioglycolate broth (Oxoid, Wesel, Germany) by the sterile scrub nurse and were transported immediately after sampling to the laboratory for culture as previously described [16, 29, 33]. Samples were incubated for 14 days or until growth was identified. If no growth was identified after 14 days of incubation, cultures were discontinued and considered negative [16, 29, 33]. Broths demonstrating bacterial growth were subcultured on appropriate agar plates. Microorganisms were identified by standard microbiologic procedures. Antibiotic susceptibility testing was performed by disk diffusion or dilution methods as described by the Clinical and Laboratory Standards Institute (CLSI) guidelines [7]. Microbiology was considered positive for PJI if the same microorganism was identified in at least two samples [4].

Tissue samples were considered histologically positive for PJI, if more than 5 neutrophils per high-power field were identified in 5 high-power fields observed from histologic analysis of periprosthetic tissue at 400 magnification [25, 26].

The diagnosis of PJI was accepted when any of the following conditions was fulfilled (1) the same microorganism was identified in at least two separate cultures or if (2) growth of one microorganism was observed in at least one culture and histopathology was positive for PJI (Krenn Morawitz classification), or (3) if four of the six minor Musculoskeletal Infection Society (MSIS) criteria were met [2, 11, 19, 22, 23, 25, 26]. Growth of a microorganism in one culture alone without histopathologic signs of an infection was regarded as a contamination [32].

Perioperative antibiotics were not administered before all samples had been taken.

The study was performed in accordance with the latest version of the Declaration of Helsinki and received ethical approval from the local ethics board (PV7213). All included patients gave written informed consent.

Sensitivity, specificity, positive predictive value (PPV), negative predictive value (NPV) and accuracy of each diagnostic method, i.e., aspiration, microbiologic examination and histologic examination from the open-biopsy procedure compared with the definitive result obtained from the revision operation, were determined [15]. Statistical analysis was performed using the statistics package SPSS version 23.0 (Version 23.0; SPSS, Chicago, IL, USA).

## Results

Thirty-two patients were included in this study (Fig. 1). The average age at THA implantation was 65.1 ± 14.5 years of age (Table 1). Seventeen cases had a primary THA and fifteen patients had already undergone a previous revision THA. All patients complained of periprosthetic localized pain. Radiologic signs of loosening were detectable in 60% of the cases (*n* = 19). The age of the patients at the time of revision surgery was 69.5 ± 13.5 years and THA survival was 4.4 ± 5.1 years on average (Table 2). Preoperative serum CRP was mildly elevated (1.9 ± 2.7 mg/dl). 17 out of 32 patients had CRP levels greater than 1.0 mg/dl with an average of 3.2 ± 3.1 mg/dl.

**Table 1:** Demographic and clinical data of 32 patients with a suspected, but not proven periprosthetic joint infection (PJI) of a total hip arthroplasty (THA)

Patients	*n* = 32
Age at implantation	65.1 ± 14.5
Age at revision (years)	69.5 ± 13.5
THA survival (years)	4.4 ± 5.1
Sex	
Male	20
Female	12
Primary THA	17 (53.2%)
Revision THA	15 (46.8%)
Pain	32 (100%)
Early Loosening (< 2 years)	19 (59.4%)
History of PJI	8 (25%)

**Table 2:** Sensitivity and specificity results of microbiology and histopathology sampling from the open incisional biopsy

	*n*
Open incisional biopsy	
Serum CRP (mg/dl)	1.9 ± 2.7
Tissue biopsy—bacteriology	32 (100%)
Number of tissue samples/case	8.7 ± 3.4
Positive tissue bacteriology (infection)	18 (56.3%)
Histology biopsy	32 (100%)
Positive histology (infection)	12 (37.5%)
Overall status: diagnosis of infection	20 (62.5%)
Revision surgery	
Serum CRP (mg/dl)	2.8 ± 4.8
Tissue biopsy—bacteriology	31 (96.9%)
Number of tissue samples/case	7.9 ± 3.1
Positive tissue bacteriology (infection)	15 (48.4%)
Histology biopsy	29 (90.6%)
Positive histology (infection)	13 (44.8%)
Overall status: diagnosis of infection	17 (53.1%)

Twenty cases were diagnosed with PJI after histological and microbiological analyses of the open incisional biopsy specimens (Fig. 1). Eighteen patients had at least two positive cultures with the same microorganism. The other two cases were classified as infected due to a combination of single positive culture, positive histology, elevated CRP and intra-operative purulence [25, 26]. No complications were observed during or after OIB as a consequence of the procedure.

Revision surgery was consequently performed as a two-stage septic revision in 20 cases (62.5%) and as a primarily aseptic single-stage revision in 12 cases (37.5%) (Fig. 1). Revision surgery with THA explanation during either one-stage or two-stage revision resulted in the final diagnosis of PJI in 17 of the 32 revised cases (53.1%). Of these 17 final diagnoses of PJI, fourteen cases had at least two positive cultures with the same microorganism and three patients fulfilled infection criteria according to the MSIS Definition of PJI [26] by a combination of minimum four minor MSIS criteria factors [25, 26].

Thus, six cases diagnosed with PJI after biopsy were not confirmed by tissue analyses from the definitive revision surgery and were therefore classified as being false positives after biopsy. Three cases were identified as PJI positive by tissue samples from single-stage revision surgery, but had not been identified by tissue biopsy samples after biopsy and thus were false negatives.

The biopsy procedure generated 12 true positives for microbiology and 9 true positives for histopathology (Table 3). Overall, sensitivity of the open biopsy was 80% (95% CI 66–94%) for microbiology of tissue samples and 69% (95% CI 53–85%) for histopathology. Specificity was 94% (95% CI 85–102%) for histopathology and 69% (95% CI 53–85%) for microbiology of tissue samples.

**Table 3:** Diagnostic accuracy of microbiology and histopathology analyses regarding the definitive diagnosis of PJI with a confidence intervals (CI) of 95%

	Microbiology^$^	Histopathology^#^	Combination^§^
Open incisional biopsy results			
True positives (*n*)	12	9	14
True negatives (*n*)	11	15	9
False positives (*n*)	5	1	6
False negatives (*n*)	3	4	3
Missing data (*n*)	1	3	0
Sensitivity (95% CI)	80 (66–94%)	69.2 (53–85%)	82.4 (69–96%)
Specificity (95% CI)	68.8 (53–85%)	93.8 (85–102%)	60 (43–77%)
Positive predictive value (95% CI)	70.6 (55–86%)	90 (80–100%)	70 (54–86%)
Negative predictive value (95% CI)	78.6 (64–93%)	78.9 (65–93%)	75 (60–90%)
Accuracy (95% CI)	74.2 (59–89%)	82.8 (70–96%)	71.9 (56–86%)

(^$^ taken at open incisional biopsy and compared with microbiology at revision surgery; ^#^ taken at open incisional biopsy and compared with histology at revision surgery; ^§^ total: combination of microbiology and histopathology)

The calculated PPV was 90% (95% CI 80–100%) for histopathology and 71% (95% CI 55–86%) for microbiology of tissue samples. The combined microbiology and histopathology demonstrated a sensitivity and specificity of 82% (95% CI 69–96%) and 60% (95% CI 43–77%). The PPV for the combination was 70% (95% CI 54–86%).

The majority of microorganisms identified during either procedure were staphylococcus epidermidis and cutibacterium acnes (Table 4). Since changes occurred in the growth of microorganisms between the two surgical procedures, the overall accuracy of both specimen collections was only 72% (95% CI 56–86%).

**Table 4:** Distribution of microorganisms identified during biopsy and revision procedures

Open incisional biopsy		Revision surgery	
Identified microorganism	Tissue biopsy	Identified microorganism	Tissue biopsy
	Number of cases with detected respective microorganisms		Number of cases with detected respective microorganisms
*Staphylococcus epidermidis*	9	*Staphylococcus epidermidis*	8
*Cutibacterium* acnes	8	*Cutiibacterium* acnes	7
*Staphylococcus capitis*	2	*Staphylococcus saccharolyticus*	2
*Staphylococcus saccharolyticus*	2	*Staphylococcus aureus*	1
*Staphylococcus aureus*	1	*Staphylococcus lugdunensis*	1
*Streptococcus agalactiae*	1	*Streptococcus agalactiae*	1
Total	23	Total	20

## Discussion

This study assessed the diagnostic value of an open incisional tissue biopsy from periprosthetic THA tissue in cases of suspected PJI despite prior negative microbiology from synovial joint fluid aspiration. In 32 consecutive cases which were then subsequently revised, open incisional biopsy resulted in a combined sensitivity of 82% and specificity of 60% for PJI and a PPV and NPV 70% and 75%, respectively.

The overall results demonstrate that open incisional biopsy is of limited predictive value to diagnose PJI prior to revision THA in this problematic patient population.

Based on the present data, one should be aware that open incisional biopsy with combined microbiology and histology analyses results in 18.8% false positives (6/32) and about 9% false negatives (3/32).

Given the approximate 9% false negatives (type II error), in this particular group of patients, it seems reasonable to extend calculated broad-spectrum antibiotic treatment until specimens taken during revision surgery come back negative from culture, even if the diagnosis of PJI has not been confirmed by any method prior to revision.

Eighteen percent false positives (type I error) from the biopsy should lead to critical reflection of the procedure as such. Based on these findings, the risks of the procedure may outweigh the benefits since it may lead to overtreatment in about 20% of revision cases in that either too many antibiotics are given for too long a period of time or in that a two-stage revision is initiated in cases where an aseptic one-stage would have been adequate.

As an alternative, less invasive methods of tissue sampling could be considered, such as arthroscopy or mini-incision with blind biopsy [1, 11, 28]. Fink and colleagues reported a combined sensitivity of 82% and specificity of 98% with a PPV of 97% for mini-incision biopsy [11] but the patient population in that study was fundamentally different from the present study as they had included every single consecutive revision case (i.e., septic, aseptic, easy to diagnose and difficult to diagnose) and not just the difficult-to-diagnose chronic low-grade infection with prior negative aspiration results [11].

Pohlig et al. reported results for the combination of bacteriology and histology with an arthroscopic biopsy technique. They described a sensitivity of 87.5%, a specificity of 100% and an accuracy of 95% [28]. Inclusion criteria were fundamentally different from the present study, including only cases with a previous PJI and elevated ESR or CRP, a suspicious cell count of the synovial fluid and early radiographic signs of loosening or persistent pain.

Another study analyzing the open biopsy method was performed by Klaber et al., they were performing open biopsies in cases of PJI suspicion after 2 negative joint fluid aspirations in a heterogenous group of THA and total knee arthroplasty (TKA) cases [17]. Their overall yield for sensitivity and specificity was 69.35% and 89.06%, respectively. The sensitivity (87.50%) and specificity (95.24%) levels for the THA subgroup were higher than in the current study, but a more detailed subgroup analysis was not available. Their conclusion of the study was that OB is a valid tool for pre-revision assessment particularly as their preferred method is a one-stage exchange surgery [17]. The one-stage exchange method showed in a systematic review and meta-analysis a lower pooled reinfection rate (5.7%) for one-stage hip revision arthroplasties when compared to the pooled reinfection rate of 8.4% in two-stage exchanges [13]. However, this review did have significantly less pooled data in the one-stage exchange group and therefore comparison is slightly skewed. Furthermore, they did not look at biopsy techniques, but what one can gather from above described literature and sampling techniques is that tissue biopsies help identifying a potential pathogen and improve treatment management [11, 13, 17, 28].

In order to carry out an optimal comparison between sampling methods (OIB, mini-incision with blind biopsy and arthroscopic biopsy), a prospective randomized study with defined inclusion and exclusion criteria for a specific patient group with analyses of all samples in the same laboratories would be needed. Furthermore, according to the literature, there is an infection risk of 2–6.8% with any procedure in orthopedic surgery [5]. Risk factors for PJI include male gender, hybrid fixation, cement without antibiotics and inflammatory diseases [8, 31]. The potential benefits of OIB have to be outweighed against the risks such as infection introduced by OIB, wound healing complications and side effects of anesthesia. In this study, we did not observe adverse events by the procedure itself.

Other methods to consider are intra-operative fast tests such as leukocyte-esterase or alpha defensin, using intra-operative obtained synovial fluid [2]. However, their use is limited when synovial fluid is contaminated with blood and sensitivity and specificity of these tests are particularly limited to detect low-grade infections [9, 18, 30].

However, based on the present data and the published results from less invasive procedures, we put forward the hypothesis that OIB is likely to be dispensable and does not need to be performed any more. Potential risks may outweigh the potential benefits. At least we do not have any indication of diagnostic superiority of OIB over other procedures of tissue sampling in this patient population. Thus, justification of OIB is at least questionable.

Nonetheless, the preoperative biopsy may be useful in the pre-revision work-up of suspected PJI, but the combination of the microbial-cultured biopsy and histopathology was of a much higher diagnostic value than the individual use as demonstrated by Li et al. [20]. Further, their study demonstrated a lack in a standardized biopsy process and thus it does not appear to have an advantage over the synovial fluid culture. Thus, the combination of multiple tests (eg. synovial fluid culture, leukocyte-esterase, alpha-defensin, biopsy, etc.) should be considered as this will help identifying a true PJI.

There are some limitations of the study. First, it is a retrospective study and thus not all aspects of pre-analytics can be fully controlled. Second, several surgeons contributed to the case series in our institution. Third, in some cases, only one tissue sample was available for histology. This is a weakness since for the reliable diagnosis of PJI, several tissue samples from different locations and in particular from the implant-bone interface, should be obtained [4, 27]. On the other hand, based on the present results it was microbiology and not histology and it was a high percentage of false positives and not false negatives that turned out to be the weak link in the procedure. However, none of these limitations is likely to have changed results significantly.

Strengths of this study include (i) tissue sample collection was done in the same way by all surgeons throughout the study period; samples were transferred to sterile containers with broth at the operating table, (ii) the microbiology and histopathology departments have been the same throughout the study period and did not change their standard analytics (iii) all samples were cultured for a minimum of 14 days until they were considered negative.

We therefore think that the current results are reliable and reproducible and thus represent a first step toward scientific evaluation of the open incisional biopsy procedure. Based on the present results we suggest that performing an open incisional biopsy requires very thorough justification, if performed at all.

## Conclusions

In conclusion, the diagnostic value of open incisional biopsy in problematic cases with suspected chronic low-grade THA PJI, despite synovial fluid-negative findings, is of limited diagnostic value. About 20% false positives and 9% false negatives should be expected. It is advantageous to define the causative pathogen prior potentially septic revision THA, but surgeons should be aware of the limitations and also potential risks of open incisional biopsy. As long as proven otherwise, alternative less invasive sampling methods, such as arthroscopy or mini-incision biopsy, should possibly be preferred in this problematic patient population.

## Data Availability

The data are not publicly available due to data containing information that could compromise research participant privacy/consent.
